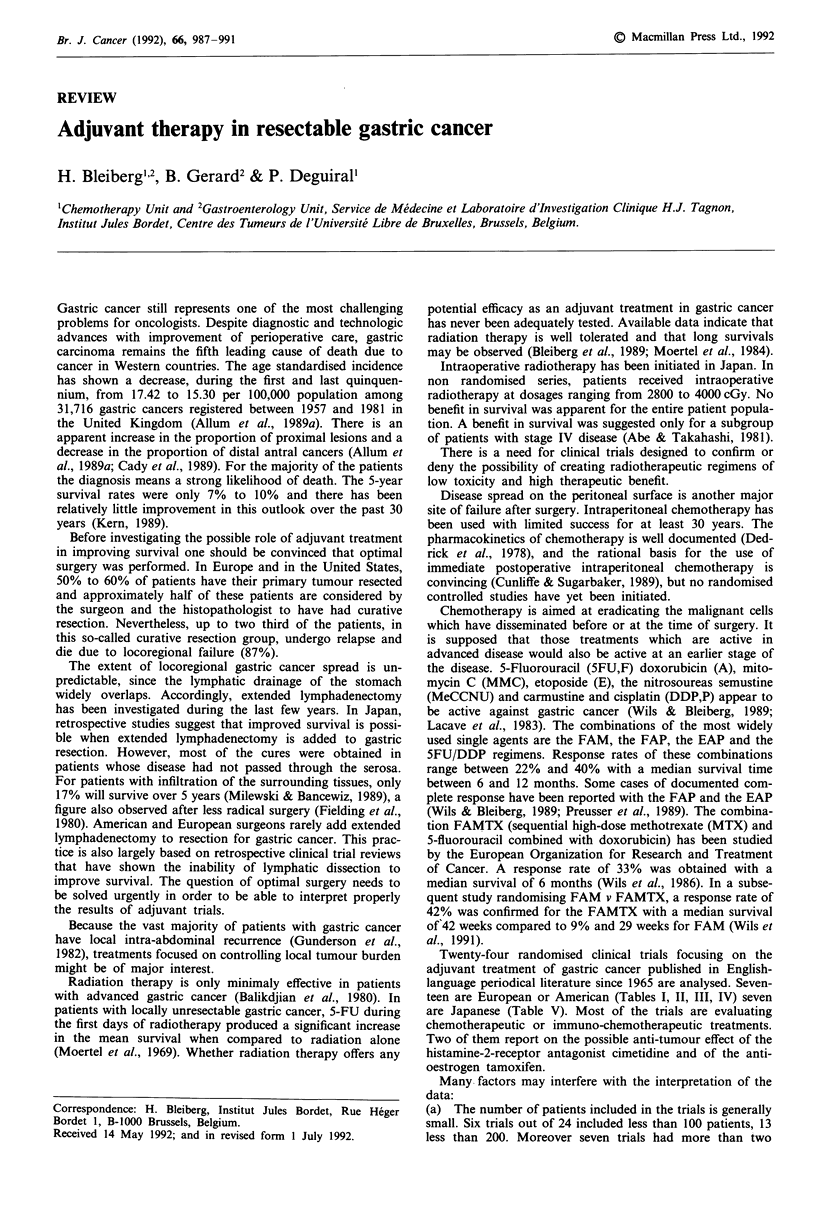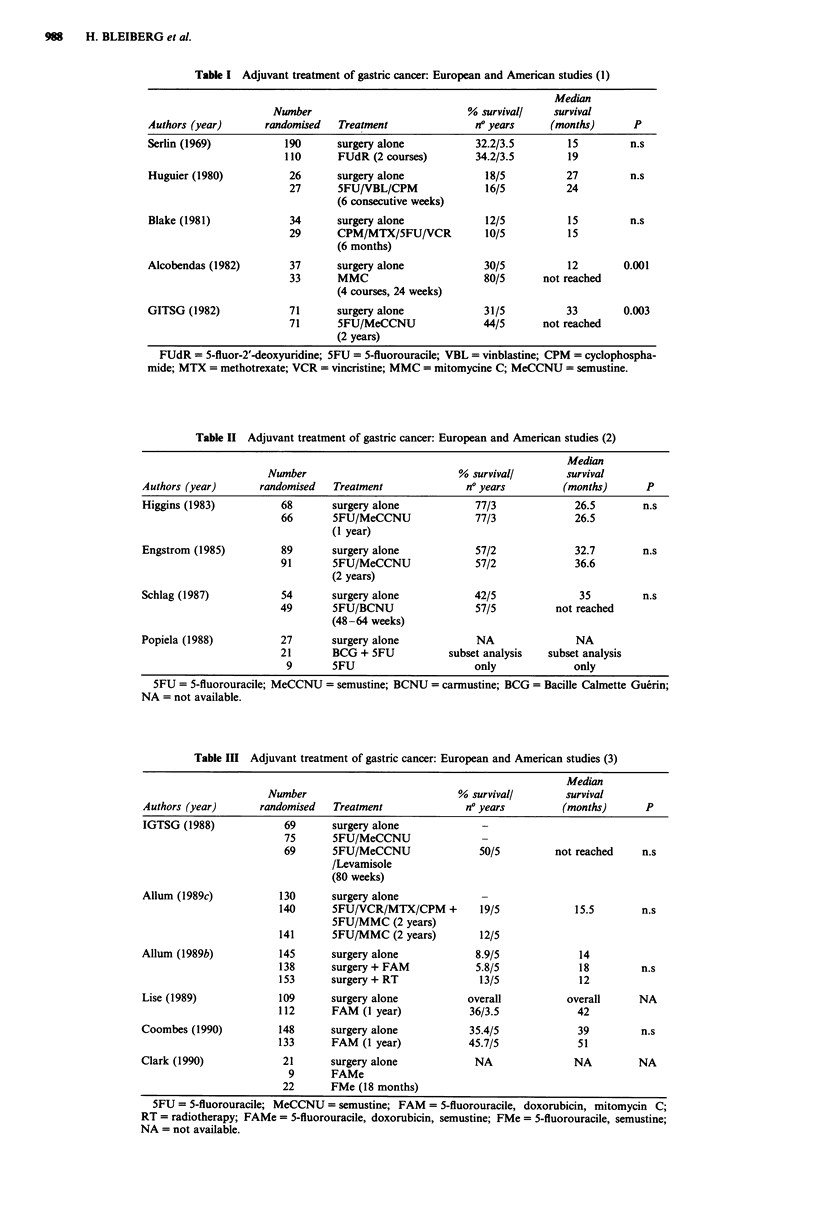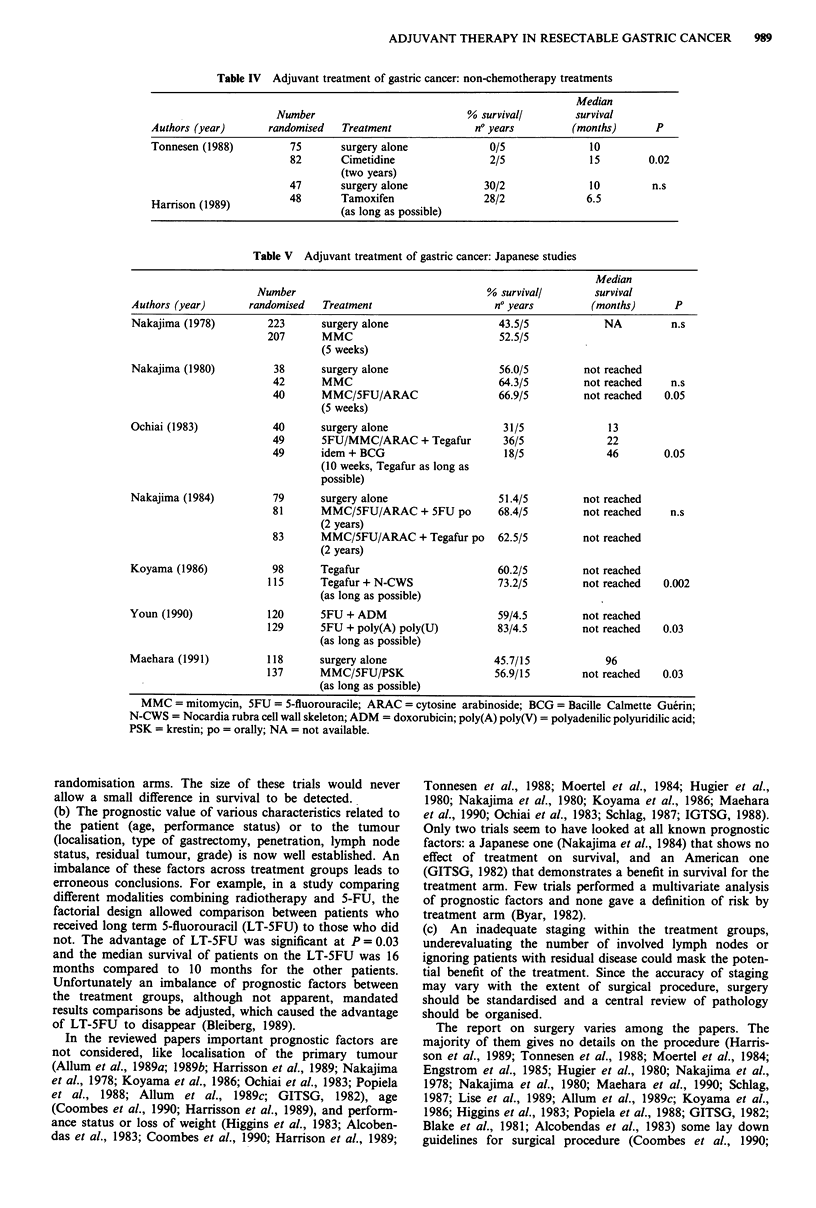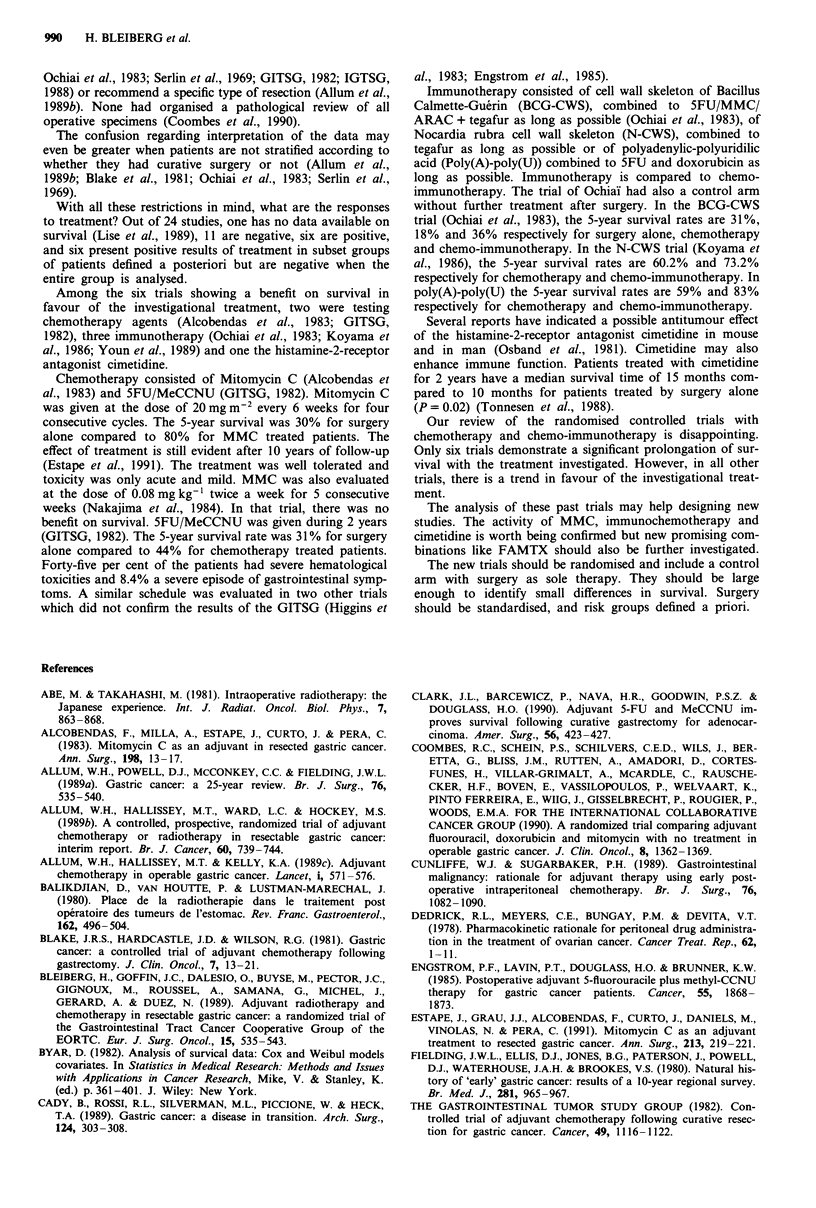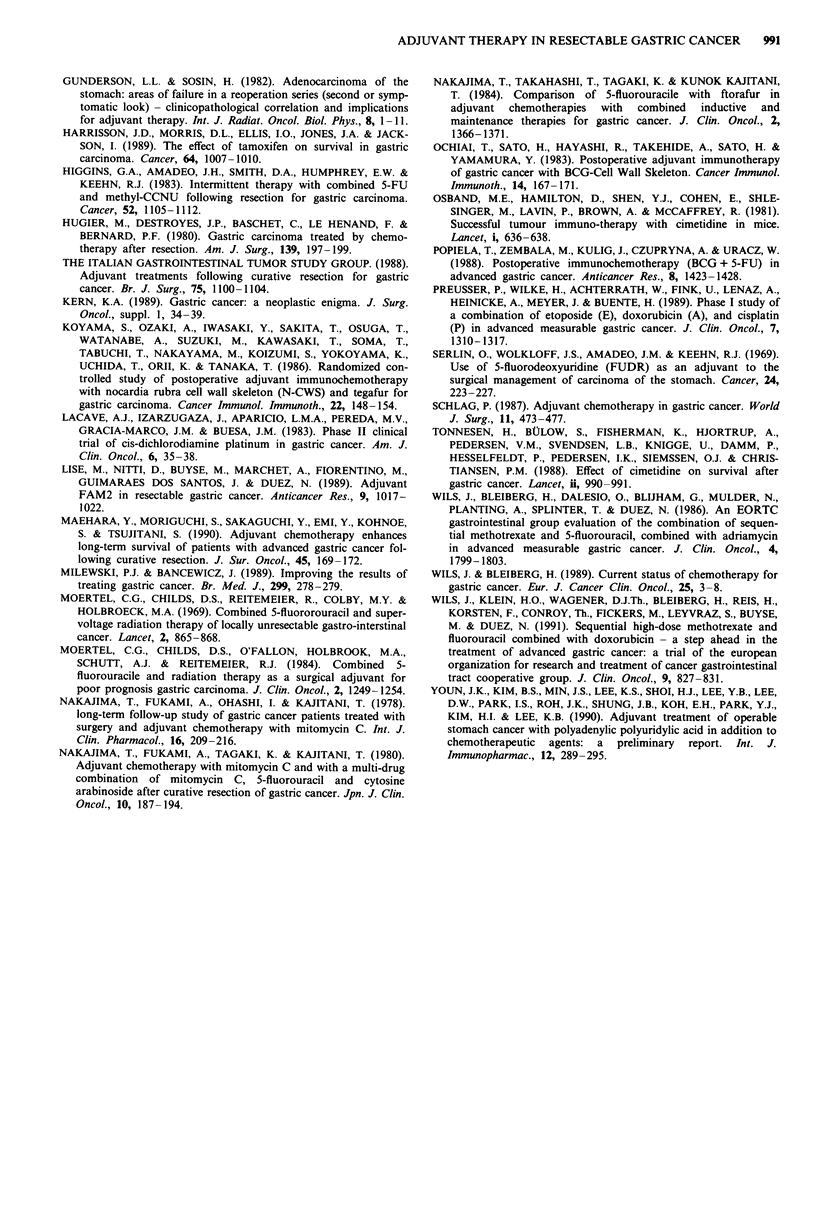# Adjuvant therapy in resectable gastric cancer.

**DOI:** 10.1038/bjc.1992.399

**Published:** 1992-12

**Authors:** H. Bleiberg, B. Gerard, P. Deguiral

**Affiliations:** Chemotherapy Unit, Institut Jules Bordet, Centre des Tumeurs de l'Université Libre de Bruxelles, Belgium.


Br. J. Cancer (1992), 66, 987–991 © Macmillan Press Ltd., 1992

REVIEW

### Adjuvant therapy in resectable gastric cancer

H. Bleiberg[1,2], B. Gerard[2] & P. Deguiral[1]

[1]*Chemotherapy Unit and* [2]*Gastroenterology Unit, Service de Médecine et Laboratoire d'Investigation Clinique H.J. Tagnon, Institut Jules Bordet, Centre des Tumeurs de l'Université Libre de Bruxelles, Brussels, Belgium.*

Gastric cancer still represents one of the most challenging problems for oncologists. Despite diagnostic and technologic advances with improvement of perioperative care, gastric carcinoma remains the fifth leading cause of death due to cancer in Western countries. The age standardised incidence has shown a decrease, during the first and last quinquennium, from 17.42 to 15.30 per 100,000 population among 31,716 gastric cancers registered between 1957 and 1981 in the United Kingdom (Allum *et al.*, 1989*a*). There is an apparent increase in the proportion of proximal lesions and a decrease in the proportion of distal antral cancers (Allum *et al.*, 1989*a*; Cady *et al.*, 1989). For the majority of the patients the diagnosis means a strong likelihood of death. The 5-year survival rates were only 7% to 10% and there has been relatively little improvement in this outlook over the past 30 years (Kern, 1989).

Before investigating the possible role of adjuvant treatment in improving survival one should be convinced that optimal surgery was performed. In Europe and in the United States, 50% to 60% of patients have their primary tumour resected and approximately half of these patients are considered by the surgeon and the histopathologist to have had curative resection. Nevertheless, up to two third of the patients, in this so-called curative resection group, undergo relapse and die due to locoregional failure (87%).

The extent of locoregional gastric cancer spread is unpredictable, since the lymphatic drainage of the stomach widely overlaps. Accordingly, extended lymphadenectomy has been investigated during the last few years. In Japan, retrospective studies suggest that improved survival is possible when extended lymphadenectomy is added to gastric resection. However, most of the cures were obtained in patients whose disease had not passed through the serosa. For patients with infiltration of the surrounding tissues, only 17% will survive over 5 years (Milewski & Bancewiz, 1989), a figure also observed after less radical surgery (Fielding *et al.*, 1980). American and European surgeons rarely add extended lymphadenectomy to resection for gastric cancer. This practice is also largely based on retrospective clinical trial reviews that have shown the inability of lymphatic dissection to improve survival. The question of optimal surgery needs to be solved urgently in order to be able to interpret properly the results of adjuvant trials.

Because the vast majority of patients with gastric cancer have local intra-abdominal recurrence (Gunderson *et al.*, 1982), treatments focused on controlling local tumour burden might be of major interest.

Radiation therapy is only minimaly effective in patients with advanced gastric cancer (Balikdjian *et al.*, 1980). In patients with locally unresectable gastric cancer, 5-FU during the first days of radiotherapy produced a significant increase in the mean survival when compared to radiation alone (Moertel *et al.*, 1969). Whether radiation therapy offers any potential efficacy as an adjuvant treatment in gastric cancer has never been adequately tested. Available data indicate that radiation therapy is well tolerated and that long survivals may be observed (Bleiberg *et al.*, 1989; Moertel *et al.*, 1984).

Intraoperative radiotherapy has been initiated in Japan. In non randomised series, patients received intraoperative radiotherapy at dosages ranging from 2800 to 4000 cGy. No benefit in survival was apparent for the entire patient population. A benefit in survival was suggested only for a subgroup of patients with stage IV disease (Abe & Takahashi, 1981).

There is a need for clinical trials designed to confirm or deny the possibility of creating radiotherapeutic regimens of low toxicity and high therapeutic benefit.

Disease spread on the peritoneal surface is another major site of failure after surgery. Intraperitoneal chemotherapy has been used with limited success for at least 30 years. The pharmacokinetics of chemotherapy is well documented (Dedrick *et al.*, 1978), and the rational basis for the use of immediate postoperative intraperitoneal chemotherapy is convincing (Cunliffe & Sugarbaker, 1989), but no randomised controlled studies have yet been initiated.

Chemotherapy is aimed at eradicating the malignant cells which have disseminated before or at the time of surgery. It is supposed that those treatments which are active in advanced disease would also be active at an earlier stage of the disease. 5-Fluorouracil (5FU,F) doxorubicin (A), mitomycin C (MMC), etoposide (E), the nitrosoureas semustine (MeCCNU) and carmustine and cisplatin (DDP,P) appear to be active against gastric cancer (Wils & Bleiberg, 1989; Lacave *et al.*, 1983). The combinations of the most widely used single agents are the FAM, the FAP, the EAP and the 5FU/DDP regimens. Response rates of these combinations range between 22% and 40% with a median survival time between 6 and 12 months. Some cases of documented complete response have been reported with the FAP and the EAP (Wils & Bleiberg, 1989; Preusser *et al.*, 1989). The combination FAMTX (sequential high-dose methotrexate (MTX) and 5-fluorouracil combined with doxorubicin) has been studied by the European Organization for Research and Treatment of Cancer. A response rate of 33% was obtained with a median survival of 6 months (Wils *et al.*, 1986). In a subsequent study randomising FAM *v* FAMTX, a response rate of 42% was confirmed for the FAMTX with a median survival of 42 weeks compared to 9% and 29 weeks for FAM (Wils *et al.*, 1991).

Twenty-four randomised clinical trials focusing on the adjuvant treatment of gastric cancer published in English-language periodical literature since 1965 are analysed. Seventeen are European or American (Tables I, II, III, IV) seven are Japanese (Table V). Most of the trials are evaluating chemotherapeutic or immuno-chemotherapeutic treatments. Two of them report on the possible anti-tumour effect of the histamine-2-receptor antagonist cimetidine and of the anti-oestrogen tamoxifen.

Many factors may interfere with the interpretation of the data:

(a) The number of patients included in the trials is generally small. Six trials out of 24 included less than 100 patients, 13 less than 200. Moreover seven trials had more than two

Correspondence: H. Bleiberg, Institut Jules Bordet, Rue Héger Bordet 1, B-1000 Brussels, Belgium.
Received 14 May 1992; and in revised form 1 July 1992.

**Table I** Adjuvant treatment of gastric cancer: European and American studies (1)

| *Authors (year)* | *Number randomised* | *Treatment* | *% survival/ n° years* | *Median survival (months)* | *P* |
|---|---|---|---|---|---|
| Serlin (1969) | 190 | surgery alone | 32.2/3.5 | 15 | n.s |
| | 110 | FUdR (2 courses) | 34.2/3.5 | 19 | |
| Huguier (1980) | 26 | surgery alone | 18/5 | 27 | n.s |
| | 27 | 5FU/VBL/CPM (6 consecutive weeks) | 16/5 | 24 | |
| Blake (1981) | 34 | surgery alone | 12/5 | 15 | n.s |
| | 29 | CPM/MTX/5FU/VCR (6 months) | 10/5 | 15 | |
| Alcobendas (1982) | 37 | surgery alone | 30/5 | 12 | 0.001 |
| | 33 | MMC (4 courses, 24 weeks) | 80/5 | not reached | |
| GITSG (1982) | 71 | surgery alone | 31/5 | 33 | 0.003 |
| | 71 | 5FU/MeCCNU (2 years) | 44/5 | not reached | |

FUdR = 5-fluor-2'-deoxyuridine; 5FU = 5-fluorouracile; VBL = vinblastine; CPM = cyclophosphamide; MTX = methotrexate; VCR = vincristine; MMC = mitomycine C; MeCCNU = semustine.

**Table II** Adjuvant treatment of gastric cancer: European and American studies (2)

| *Authors (year)* | *Number randomised* | *Treatment* | *% survival/ n° years* | *Median survival (months)* | *P* |
|---|---|---|---|---|---|
| Higgins (1983) | 68 | surgery alone | 77/3 | 26.5 | n.s |
| | 66 | 5FU/MeCCNU (1 year) | 77/3 | 26.5 | |
| Engstrom (1985) | 89 | surgery alone | 57/2 | 32.7 | n.s |
| | 91 | 5FU/MeCCNU (2 years) | 57/2 | 36.6 | |
| Schlag (1987) | 54 | surgery alone | 42/5 | 35 | n.s |
| | 49 | 5FU/BCNU (48–64 weeks) | 57/5 | not reached | |
| Popiela (1988) | 27 | surgery alone | NA | NA | |
| | 21 | BCG + 5FU | subset analysis | subset analysis | |
| | 9 | 5FU | only | only | |

5FU = 5-fluorouracile; MeCCNU = semustine; BCNU = carmustine; BCG = Bacille Calmette Guérin; NA = not available.

**Table III** Adjuvant treatment of gastric cancer: European and American studies (3)

| *Authors (year)* | *Number randomised* | *Treatment* | *% survival/ n° years* | *Median survival (months)* | *P* |
|---|---|---|---|---|---|
| IGTSG (1988) | 69 | surgery alone | – | | |
| | 75 | 5FU/MeCCNU | – | | |
| | 69 | 5FU/MeCCNU /Levamisole (80 weeks) | 50/5 | not reached | n.s |
| Allum (1989*c*) | 130 | surgery alone | – | | |
| | 140 | 5FU/VCR/MTX/CPM + 5FU/MMC (2 years) | 19/5 | 15.5 | n.s |
| | 141 | 5FU/MMC (2 years) | 12/5 | | |
| Allum (1989*b*) | 145 | surgery alone | 8.9/5 | 14 | |
| | 138 | surgery + FAM | 5.8/5 | 18 | n.s |
| | 153 | surgery + RT | 13/5 | 12 | |
| Lise (1989) | 109 | surgery alone | overall | overall | NA |
| | 112 | FAM (1 year) | 36/3.5 | 42 | |
| Coombes (1990) | 148 | surgery alone | 35.4/5 | 39 | n.s |
| | 133 | FAM (1 year) | 45.7/5 | 51 | |
| Clark (1990) | 21 | surgery alone | NA | NA | NA |
| | 9 | FAMe | | | |
| | 22 | FMe (18 months) | | | |

5FU = 5-fluorouracile; MeCCNU = semustine; FAM = 5-fluorouracile, doxorubicin, mitomycin C; RT = radiotherapy; FAMe = 5-fluorouracile, doxorubicin, semustine; FMe = 5-fluorouracile, semustine; NA = not available.

**Table IV** Adjuvant treatment of gastric cancer: non-chemotherapy treatments

| *Authors (year)* | *Number randomised* | *Treatment* | *% survival/ n° years* | *Median survival (months)* | *P* |
|---|---|---|---|---|---|
| Tonnesen (1988) | 75 | surgery alone | 0/5 | 10 | |
| | 82 | Cimetidine (two years) | 2/5 | 15 | 0.02 |
| Harrison (1989) | 47 | surgery alone | 30/2 | 10 | n.s |
| | 48 | Tamoxifen (as long as possible) | 28/2 | 6.5 | |

**Table V** Adjuvant treatment of gastric cancer: Japanese studies

| *Authors (year)* | *Number randomised* | *Treatment* | *% survival/ n° years* | *Median survival (months)* | *P* |
|---|---|---|---|---|---|
| Nakajima (1978) | 223 | surgery alone | 43.5/5 | NA | n.s |
| | 207 | MMC (5 weeks) | 52.5/5 | | |
| Nakajima (1980) | 38 | surgery alone | 56.0/5 | not reached | |
| | 42 | MMC | 64.3/5 | not reached | n.s |
| | 40 | MMC/5FU/ARAC (5 weeks) | 66.9/5 | not reached | 0.05 |
| Ochiai (1983) | 40 | surgery alone | 31/5 | 13 | |
| | 49 | 5FU/MMC/ARAC + Tegafur | 36/5 | 22 | |
| | 49 | idem + BCG (10 weeks, Tegafur as long as possible) | 18/5 | 46 | 0.05 |
| Nakajima (1984) | 79 | surgery alone | 51.4/5 | not reached | |
| | 81 | MMC/5FU/ARAC + 5FU po (2 years) | 68.4/5 | not reached | n.s |
| | 83 | MMC/5FU/ARAC + Tegafur po (2 years) | 62.5/5 | not reached | |
| Koyama (1986) | 98 | Tegafur | 60.2/5 | not reached | |
| | 115 | Tegafur + N-CWS (as long as possible) | 73.2/5 | not reached | 0.002 |
| Youn (1990) | 120 | 5FU + ADM | 59/4.5 | not reached | |
| | 129 | 5FU + poly(A) poly(U) (as long as possible) | 83/4.5 | not reached | 0.03 |
| Maehara (1991) | 118 | surgery alone | 45.7/15 | 96 | |
| | 137 | MMC/5FU/PSK (as long as possible) | 56.9/15 | not reached | 0.03 |

MMC = mitomycin, 5FU = 5-fluorouracile; ARAC = cytosine arabinoside; BCG = Bacille Calmette Guérin; N-CWS = Nocardia rubra cell wall skeleton; ADM = doxorubicin; poly(A) poly(V) = polyadenilic polyuridilic acid; PSK = krestin; po = orally; NA = not available.

randomisation arms. The size of these trials would never allow a small difference in survival to be detected.

(b) The prognostic value of various characteristics related to the patient (age, performance status) or to the tumour (localisation, type of gastrectomy, penetration, lymph node status, residual tumour, grade) is now well established. An imbalance of these factors across treatment groups leads to erroneous conclusions. For example, in a study comparing different modalities combining radiotherapy and 5-FU, the factorial design allowed comparison between patients who received long term 5-fluorouracil (LT-5FU) to those who did not. The advantage of LT-5FU was significant at $P = 0.03$ and the median survival of patients on the LT-5FU was 16 months compared to 10 months for the other patients. Unfortunately an imbalance of prognostic factors between the treatment groups, although not apparent, mandated results comparisons be adjusted, which caused the advantage of LT-5FU to disappear (Bleiberg, 1989).

In the reviewed papers important prognostic factors are not considered, like localisation of the primary tumour (Allum *et al.*, 1989*a*; 1989*b*; Harrisson *et al.*, 1989; Nakajima *et al.*, 1978; Koyama *et al.*, 1986; Ochiai *et al.*, 1983; Popiela *et al.*, 1988; Allum *et al.*, 1989*c*; GITSG, 1982), age (Coombes *et al.*, 1990; Harrisson *et al.*, 1989), and performance status or loss of weight (Higgins *et al.*, 1983; Alcobendas *et al.*, 1983; Coombes *et al.*, 1990; Harrison *et al.*, 1989; Tonnesen *et al.*, 1988; Moertel *et al.*, 1984; Hugier *et al.*, 1980; Nakajima *et al.*, 1980; Koyama *et al.*, 1986; Maehara *et al.*, 1990; Ochiai *et al.*, 1983; Schlag, 1987; IGTSG, 1988). Only two trials seem to have looked at all known prognostic factors: a Japanese one (Nakajima *et al.*, 1984) that shows no effect of treatment on survival, and an American one (GITSG, 1982) that demonstrates a benefit in survival for the treatment arm. Few trials performed a multivariate analysis of prognostic factors and none gave a definition of risk by treatment arm (Byar, 1982).

(c) An inadequate staging within the treatment groups, underevaluating the number of involved lymph nodes or ignoring patients with residual disease could mask the potential benefit of the treatment. Since the accuracy of staging may vary with the extent of surgical procedure, surgery should be standardised and a central review of pathology should be organised.

The report on surgery varies among the papers. The majority of them gives no details on the procedure (Harrisson *et al.*, 1989; Tonnesen *et al.*, 1988; Moertel *et al.*, 1984; Engstrom *et al.*, 1985; Hugier *et al.*, 1980; Nakajima *et al.*, 1978; Nakajima *et al.*, 1980; Maehara *et al.*, 1990; Schlag, 1987; Lise *et al.*, 1989; Allum *et al.*, 1989*c*; Koyama *et al.*, 1986; Higgins *et al.*, 1983; Popiela *et al.*, 1988; GITSG, 1982; Blake *et al.*, 1981; Alcobendas *et al.*, 1983) some lay down guidelines for surgical procedure (Coombes *et al.*, 1990;

Ochiai *et al.*, 1983; Serlin *et al.*, 1969; GITSG, 1982; IGTSG, 1988) or recommend a specific type of resection (Allum *et al.*, 1989*b*). None had organised a pathological review of all operative specimens (Coombes *et al.*, 1990).

The confusion regarding interpretation of the data may even be greater when patients are not stratified according to whether they had curative surgery or not (Allum *et al.*, 1989*b*; Blake *et al.*, 1981; Ochiai *et al.*, 1983; Serlin *et al.*, 1969).

With all these restrictions in mind, what are the responses to treatment? Out of 24 studies, one has no data available on survival (Lise *et al.*, 1989), 11 are negative, six are positive, and six present positive results of treatment in subset groups of patients defined a posteriori but are negative when the entire group is analysed.

Among the six trials showing a benefit on survival in favour of the investigational treatment, two were testing chemotherapy agents (Alcobendas *et al.*, 1983; GITSG, 1982), three immunotherapy (Ochiai *et al.*, 1983; Koyama *et al.*, 1986; Youn *et al.*, 1989) and one the histamine-2-receptor antagonist cimetidine.

Chemotherapy consisted of Mitomycin C (Alcobendas *et al.*, 1983) and 5FU/MeCCNU (GITSG, 1982). Mitomycin C was given at the dose of $20\,\mathrm{mg\,m^{-2}}$ every 6 weeks for four consecutive cycles. The 5-year survival was 30% for surgery alone compared to 80% for MMC treated patients. The effect of treatment is still evident after 10 years of follow-up (Estape *et al.*, 1991). The treatment was well tolerated and toxicity was only acute and mild. MMC was also evaluated at the dose of $0.08\,\mathrm{mg\,kg^{-1}}$ twice a week for 5 consecutive weeks (Nakajima *et al.*, 1984). In that trial, there was no benefit on survival. 5FU/MeCCNU was given during 2 years (GITSG, 1982). The 5-year survival rate was 31% for surgery alone compared to 44% for chemotherapy treated patients. Forty-five per cent of the patients had severe hematological toxicities and 8.4% a severe episode of gastrointestinal symptoms. A similar schedule was evaluated in two other trials which did not confirm the results of the GITSG (Higgins *et al.*, 1983; Engstrom *et al.*, 1985).

Immunotherapy consisted of cell wall skeleton of Bacillus Calmette-Guérin (BCG-CWS), combined to 5FU/MMC/ARAC + tegafur as long as possible (Ochiai *et al.*, 1983), of Nocardia rubra cell wall skeleton (N-CWS), combined to tegafur as long as possible or of polyadenylic-polyuridilic acid (Poly(A)-poly(U)) combined to 5FU and doxorubicin as long as possible. Immunotherapy is compared to chemo-immunotherapy. The trial of Ochiaï had also a control arm without further treatment after surgery. In the BCG-CWS trial (Ochiai *et al.*, 1983), the 5-year survival rates are 31%, 18% and 36% respectively for surgery alone, chemotherapy and chemo-immunotherapy. In the N-CWS trial (Koyama *et al.*, 1986), the 5-year survival rates are 60.2% and 73.2% respectively for chemotherapy and chemo-immunotherapy. In poly(A)-poly(U) the 5-year survival rates are 59% and 83% respectively for chemotherapy and chemo-immunotherapy.

Several reports have indicated a possible antitumour effect of the histamine-2-receptor antagonist cimetidine in mouse and in man (Osband *et al.*, 1981). Cimetidine may also enhance immune function. Patients treated with cimetidine for 2 years have a median survival time of 15 months compared to 10 months for patients treated by surgery alone ($P = 0.02$) (Tonnesen *et al.*, 1988).

Our review of the randomised controlled trials with chemotherapy and chemo-immunotherapy is disappointing. Only six trials demonstrate a significant prolongation of survival with the treatment investigated. However, in all other trials, there is a trend in favour of the investigational treatment.

The analysis of these past trials may help designing new studies. The activity of MMC, immunochemotherapy and cimetidine is worth being confirmed but new promising combinations like FAMTX should also be further investigated.

The new trials should be randomised and include a control arm with surgery as sole therapy. They should be large enough to identify small differences in survival. Surgery should be standardised, and risk groups defined a priori.

### References

ABE, M. & TAKAHASHI, M. (1981). Intraoperative radiotherapy: the Japanese experience. *Int. J. Radiat. Oncol. Biol. Phys.*, **7**, 863–868.

ALCOBENDAS, F., MILLA, A., ESTAPE, J., CURTO, J. & PERA, C. (1983). Mitomycin C as an adjuvant in resected gastric cancer. *Ann. Surg.*, **198**, 13–17.

ALLUM, W.H., POWELL, D.J., MCCONKEY, C.C. & FIELDING, J.W.L. (1989*a*). Gastric cancer: a 25-year review. *Br. J. Surg.*, **76**, 535–540.

ALLUM, W.H., HALLISSEY, M.T., WARD, L.C. & HOCKEY, M.S. (1989*b*). A controlled, prospective, randomized trial of adjuvant chemotherapy or radiotherapy in resectable gastric cancer: interim report. *Br. J. Cancer*, **60**, 739–744.

ALLUM, W.H., HALLISSEY, M.T. & KELLY, K.A. (1989*c*). Adjuvant chemotherapy in operable gastric cancer. *Lancet*, **i**, 571–576.

BALIKDJIAN, D., VAN HOUTTE, P. & LUSTMAN-MARECHAL, J. (1980). Place de la radiotherapie dans le traitement post opèratoire des tumeurs de l'estomac. *Rev. Franc. Gastroenterol.*, **162**, 496–504.

BLAKE, J.R.S., HARDCASTLE, J.D. & WILSON, R.G. (1981). Gastric cancer: a controlled trial of adjuvant chemotherapy following gastrectomy. *J. Clin. Oncol.*, **7**, 13–21.

BLEIBERG, H., GOFFIN, J.C., DALESIO, O., BUYSE, M., PECTOR, J.C., GIGNOUX, M., ROUSSEL, A., SAMANA, G., MICHEL, J., GERARD, A. & DUEZ, N. (1989). Adjuvant radiotherapy and chemotherapy in resectable gastric cancer: a randomized trial of the Gastrointestinal Tract Cancer Cooperative Group of the EORTC. *Eur. J. Surg. Oncol.*, **15**, 535–543.

BYAR, D. (1982). Analysis of survical data: Cox and Weibul models covariates. In *Statistics in Medical Research: Methods and Issues with Applications in Cancer Research*, Mike, V. & Stanley, K. (ed.) p. 361–401. J. Wiley: New York.

CADY, B., ROSSI, R.L., SILVERMAN, M.L., PICCIONE, W. & HECK, T.A. (1989). Gastric cancer: a disease in transition. *Arch. Surg.*, **124**, 303–308.

CLARK, J.L., BARCEWICZ, P., NAVA, H.R., GOODWIN, P.S.Z. & DOUGLASS, H.O. (1990). Adjuvant 5-FU and MeCCNU improves survival following curative gastrectomy for adenocarcinoma. *Amer. Surg.*, **56**, 423–427.

COOMBES, R.C., SCHEIN, P.S., SCHILVERS, C.E.D., WILS, J., BERETTA, G., BLISS, J.M., RUTTEN, A., AMADORI, D., CORTES-FUNES, H., VILLAR-GRIMALT, A., MCARDLE, C., RAUSCHECKER, H.F., BOVEN, E., VASSILOPOULOS, P., WELVAART, K., PINTO FERREIRA, E., WIIG, J., GISSELBRECHT, P., ROUGIER, P., WOODS, E.M.A. FOR THE INTERNATIONAL COLLABORATIVE CANCER GROUP (1990). A randomized trial comparing adjuvant fluorouracil, doxorubicin and mitomycin with no treatment in operable gastric cancer. *J. Clin. Oncol.*, **8**, 1362–1369.

CUNLIFFE, W.J. & SUGARBAKER, P.H. (1989). Gastrointestinal malignancy: rationale for adjuvant therapy using early post-operative intraperitoneal chemotherapy. *Br. J. Surg.*, **76**, 1082–1090.

DEDRICK, R.L., MEYERS, C.E., BUNGAY, P.M. & DEVITA, V.T. (1978). Pharmacokinetic rationale for peritoneal drug administration in the treatment of ovarian cancer. *Cancer Treat. Rep.*, **62**, 1–11.

ENGSTROM, P.F., LAVIN, P.T., DOUGLASS, H.O. & BRUNNER, K.W. (1985). Postoperative adjuvant 5-fluorouracile plus methyl-CCNU therapy for gastric cancer patients. *Cancer*, **55**, 1868–1873.

ESTAPE, J., GRAU, J.J., ALCOBENDAS, F., CURTO, J., DANIELS, M., VINOLAS, N. & PERA, C. (1991). Mitomycin C as an adjuvant treatment to resected gastric cancer. *Ann. Surg.*, **213**, 219–221.

FIELDING, J.W.L., ELLIS, D.J., JONES, B.G., PATERSON, J., POWELL, D.J., WATERHOUSE, J.A.H. & BROOKES, V.S. (1980). Natural history of 'early' gastric cancer: results of a 10-year regional survey. *Br. Med. J.*, **281**, 965–967.

THE GASTROINTESTINAL TUMOR STUDY GROUP (1982). Controlled trial of adjuvant chemotherapy following curative resection for gastric cancer. *Cancer*, **49**, 1116–1122.

GUNDERSON, L.L. & SOSIN, H. (1982). Adenocarcinoma of the stomach: areas of failure in a reoperation series (second or symptomatic look) – clinicopathological correlation and implications for adjuvant therapy. *Int. J. Radiat. Oncol. Biol. Phys.*, **8**, 1–11.

HARRISSON, J.D., MORRIS, D.L., ELLIS, I.O., JONES, J.A. & JACKSON, I. (1989). The effect of tamoxifen on survival in gastric carcinoma. *Cancer*, **64**, 1007–1010.

HIGGINS, G.A., AMADEO, J.H., SMITH, D.A., HUMPHREY, E.W. & KEEHN, R.J. (1983). Intermittent therapy with combined 5-FU and methyl-CCNU following resection for gastric carcinoma. *Cancer*, **52**, 1105–1112.

HUGIER, M., DESTROYES, J.P., BASCHET, C., LE HENAND, F. & BERNARD, P.F. (1980). Gastric carcinoma treated by chemotherapy after resection. *Am. J. Surg.*, **139**, 197–199.

THE ITALIAN GASTROINTESTINAL TUMOR STUDY GROUP. (1988). Adjuvant treatments following curative resection for gastric cancer. *Br. J. Surg.*, **75**, 1100–1104.

KERN, K.A. (1989). Gastric cancer: a neoplastic enigma. *J. Surg. Oncol.*, suppl. 1, 34–39.

KOYAMA, S., OZAKI, A., IWASAKI, Y., SAKITA, T., OSUGA, T., WATANABE, A., SUZUKI, M., KAWASAKI, T., SOMA, T., TABUCHI, T., NAKAYAMA, M., KOIZUMI, S., YOKOYAMA, K., UCHIDA, T., ORII, K. & TANAKA, T. (1986). Randomized controlled study of postoperative adjuvant immunochemotherapy with nocardia rubra cell wall skeleton (N-CWS) and tegafur for gastric carcinoma. *Cancer Immunol. Immunoth.*, **22**, 148–154.

LACAVE, A.J., IZARZUGAZA, J., APARICIO, L.M.A., PEREDA, M.V., GRACIA-MARCO, J.M. & BUESA, J.M. (1983). Phase II clinical trial of cis-dichlorodiamine platinum in gastric cancer. *Am. J. Clin. Oncol.*, **6**, 35–38.

LISE, M., NITTI, D., BUYSE, M., MARCHET, A., FIORENTINO, M., GUIMARAES DOS SANTOS, J. & DUEZ, N. (1989). Adjuvant FAM2 in resectable gastric cancer. *Anticancer Res.*, **9**, 1017–1022.

MAEHARA, Y., MORIGUCHI, S., SAKAGUCHI, Y., EMI, Y., KOHNOE, S. & TSUJITANI, S. (1990). Adjuvant chemotherapy enhances long-term survival of patients with advanced gastric cancer following curative resection. *J. Sur. Oncol.*, **45**, 169–172.

MILEWSKI, P.J. & BANCEWICZ, J. (1989). Improving the results of treating gastric cancer. *Br. Med. J.*, **299**, 278–279.

MOERTEL, C.G., CHILDS, D.S., REITEMEIER, R., COLBY, M.Y. & HOLBROECK, M.A. (1969). Combined 5-fluororouracil and supervoltage radiation therapy of locally unresectable gastro-interstinal cancer. *Lancet*, **2**, 865–868.

MOERTEL, C.G., CHILDS, D.S., O'FALLON, HOLBROOK, M.A., SCHUTT, A.J. & REITEMEIER, R.J. (1984). Combined 5-fluorouracile and radiation therapy as a surgical adjuvant for poor prognosis gastric carcinoma. *J. Clin. Oncol.*, **2**, 1249–1254.

NAKAJIMA, T., FUKAMI, A., OHASHI, I. & KAJITANI, T. (1978). long-term follow-up study of gastric cancer patients treated with surgery and adjuvant chemotherapy with mitomycin C. *Int. J. Clin. Pharmacol.*, **16**, 209–216.

NAKAJIMA, T., FUKAMI, A., TAGAKI, K. & KAJITANI, T. (1980). Adjuvant chemotherapy with mitomycin C and with a multi-drug combination of mitomycin C, 5-fluorouracil and cytosine arabinoside after curative resection of gastric cancer. *Jpn. J. Clin. Oncol.*, **10**, 187–194.

NAKAJIMA, T., TAKAHASHI, T., TAGAKI, K. & KUNOK KAJITANI, T. (1984). Comparison of 5-fluorouracile with ftorafur in adjuvant chemotherapies with combined inductive and maintenance therapies for gastric cancer. *J. Clin. Oncol.*, **2**, 1366–1371.

OCHIAI, T., SATO, H., HAYASHI, R., TAKEHIDE, A., SATO, H. & YAMAMURA, Y. (1983). Postoperative adjuvant immunotherapy of gastric cancer with BCG-Cell Wall Skeleton. *Cancer Immunol. Immunoth.*, **14**, 167–171.

OSBAND, M.E., HAMILTON, D., SHEN, Y.J., COHEN, E., SHLESINGER, M., LAVIN, P., BROWN, A. & MCCAFFREY, R. (1981). Successful tumour immuno-therapy with cimetidine in mice. *Lancet*, **i**, 636–638.

POPIELA, T., ZEMBALA, M., KULIG, J., CZUPRYNA, A. & URACZ, W. (1988). Postoperative immunochemotherapy (BCG + 5-FU) in advanced gastric cancer. *Anticancer Res.*, **8**, 1423–1428.

PREUSSER, P., WILKE, H., ACHTERRATH, W., FINK, U., LENAZ, A., HEINICKE, A., MEYER, J. & BUENTE, H. (1989). Phase I study of a combination of etoposide (E), doxorubicin (A), and cisplatin (P) in advanced measurable gastric cancer. *J. Clin. Oncol.*, **7**, 1310–1317.

SERLIN, O., WOLKLOFF, J.S., AMADEO, J.M. & KEEHN, R.J. (1969). Use of 5-fluorodeoxyuridine (FUDR) as an adjuvant to the surgical management of carcinoma of the stomach. *Cancer*, **24**, 223–227.

SCHLAG, P. (1987). Adjuvant chemotherapy in gastric cancer. *World J. Surg.*, **11**, 473–477.

TONNESEN, H., BÜLOW, S., FISHERMAN, K., HJORTRUP, A., PEDERSEN, V.M., SVENDSEN, L.B., KNIGGE, U., DAMM, P., HESSELFELDT, P., PEDERSEN, I.K., SIEMSSEN, O.J. & CHRISTIANSEN, P.M. (1988). Effect of cimetidine on survival after gastric cancer. *Lancet*, **ii**, 990–991.

WILS, J., BLEIBERG, H., DALESIO, O., BLIJHAM, G., MULDER, N., PLANTING, A., SPLINTER, T. & DUEZ, N. (1986). An EORTC gastrointestinal group evaluation of the combination of sequential methotrexate and 5-fluorouracil, combined with adriamycin in advanced measurable gastric cancer. *J. Clin. Oncol.*, **4**, 1799–1803.

WILS, J. & BLEIBERG, H. (1989). Current status of chemotherapy for gastric cancer. *Eur. J. Cancer Clin. Oncol.*, **25**, 3–8.

WILS, J., KLEIN, H.O., WAGENER, D.J.Th., BLEIBERG, H., REIS, H., KORSTEN, F., CONROY, Th., FICKERS, M., LEYVRAZ, S., BUYSE, M. & DUEZ, N. (1991). Sequential high-dose methotrexate and fluorouracil combined with doxorubicin – a step ahead in the treatment of advanced gastric cancer: a trial of the european organization for research and treatment of cancer gastrointestinal tract cooperative group. *J. Clin. Oncol.*, **9**, 827–831.

YOUN, J.K., KIM, B.S., MIN, J.S., LEE, K.S., SHOI, H.J., LEE, Y.B., LEE, D.W., PARK, I.S., ROH, J.K., SHUNG, J.B., KOH, E.H., PARK, Y.J., KIM, H.I. & LEE, K.B. (1990). Adjuvant treatment of operable stomach cancer with polyadenylic polyuridylic acid in addition to chemotherapeutic agents: a preliminary report. *Int. J. Immunopharmac.*, **12**, 289–295.